# An analysis of network brokerage and geographic location in fifteenth-century AD Northern Iroquoia

**DOI:** 10.1371/journal.pone.0209689

**Published:** 2019-01-09

**Authors:** John P. Hart, Susan Winchell-Sweeney, Jennifer Birch

**Affiliations:** 1 Research and Collections Division, New York State Museum, 3140 Cultural Education Center, Albany, New York, United States of America; 2 Department of Anthropology, University of Georgia, Athens, Georgia, United States of America; Universidad Rey Juan Carlos, SPAIN

## Abstract

Iroquoian villagers living in present-day Jefferson County, New York, at the headwaters of the St. Lawrence River and the east shore of Lake Ontario, played important roles in regional interactions during the fifteenth century AD, as brokers linking populations on the north shore of Lake Ontario with populations in eastern New York. This study employs a social network analysis and least cost path analysis to assess the degree to which geographical location may have facilitated the brokerage positions of site clusters within pan-Iroquoian social networks. The results indicate that location was a significant factor in determining brokerage. In the sixteenth century AD, when Jefferson County was abandoned, measurable increases in social distance between other Iroquoian populations obtained. These results add to our understandings of the dynamic social landscape of fifteenth and sixteenth century AD northern Iroquoia, complementing recent analyses elsewhere of the roles played in regional interaction networks by populations located along geopolitical frontiers.

## Introduction

When Europeans began arriving in northeastern North America in the sixteenth- and seventeenth-centuries AD, they entered a social landscape that was in the process of dynamic geopolitical transformation. Over the course of the fifteenth- and sixteenth-centuries AD, Iroquoians living on the north shore of Lake Ontario in present-day Ontario moved northward toward the south shore of Georgian Bay, while at the same time coalescing into large villages and towns, forming nations, and eventually the Wendat (Huron) confederacy [1–3]. In the Finger Lakes region and Mohawk River valley of present-day New York, the formation of larger villages was also a trend, but geographical coalescence did not occur as in Ontario. Instead, discrete clusters of towns formed nations that were united as the Haudenosaunee (Iroquois) confederacy [4]. These processes of settlement aggregation and regional political realignment were part of the formation of very different Wendat and Haudenosaunee confederacy structures beginning in the sixteenth century AD, in present-day Ontario and New York, respectively [5]. In contrast to these processes of political consolidation, Iroquoian villagers living in the St. Lawrence River valley dispersed in the late fifteenth and sixteenth centuries [6,7]. While questions about the “disappearance” of St. Lawrence Iroquoian societies preoccupied archaeologists in the mid-twentieth century [8,9], current theories hold that these populations were absorbed by the aforementioned Iroquoian groups living to the north and south [2,6,7,10]. However, we are only beginning to understand the role that these populations played in the social and political landscape of fifteenth- and sixteenth-century Iroquoia, a topic to which this study contributes.

**Fig 1:**
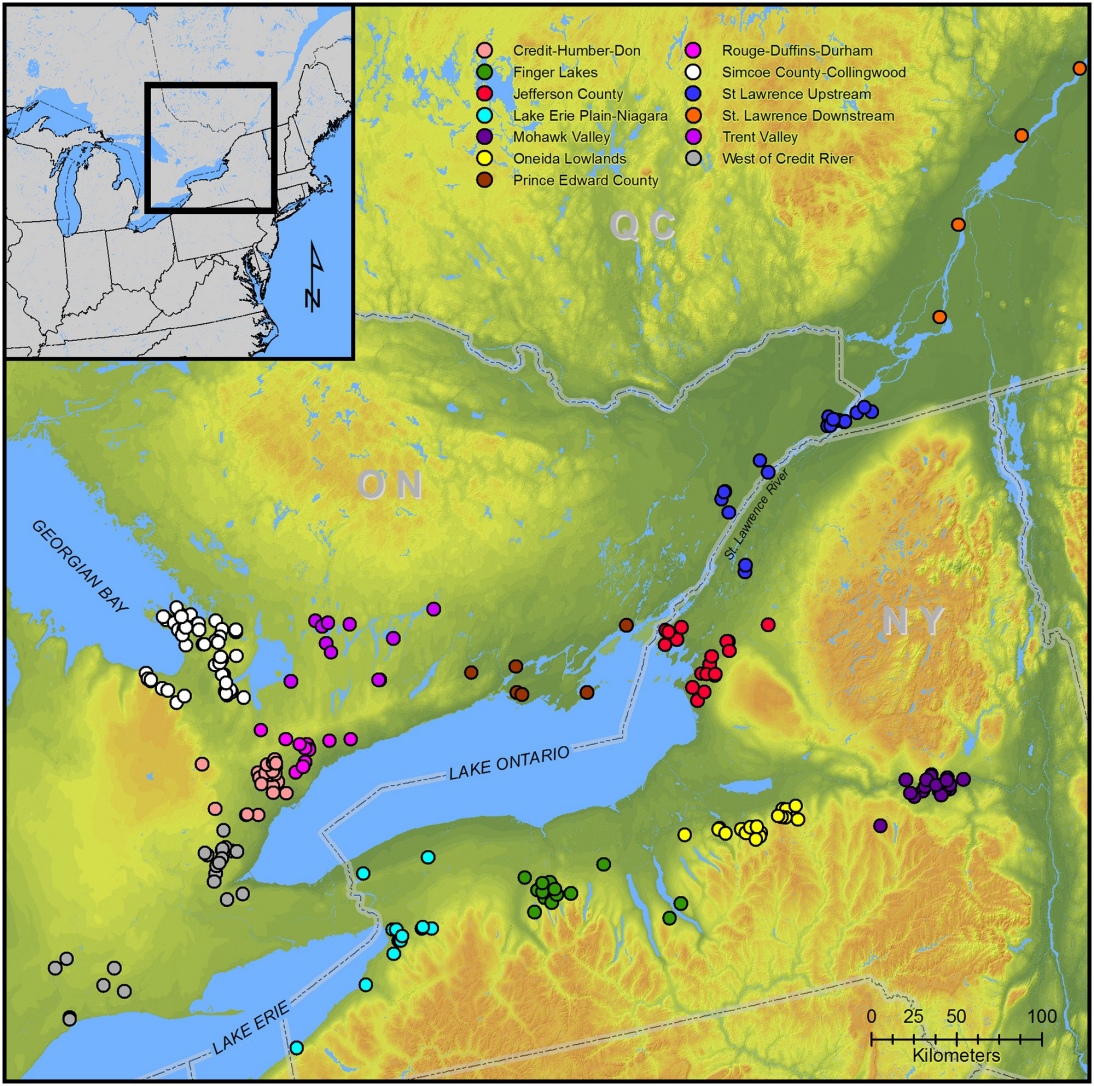
Map showing locations of sites in database keyed to clusters used in the current analyses.

It is becoming increasingly apparent that Northern Iroquoian villagers living in present-day Jefferson County, New York (hereafter JCI), at the headwaters of the St. Lawrence River and the east shore of Lake Ontario, played important roles in regional interactions during the fifteenth century AD, particularly between Iroquoians living on Lake Ontario’s north shore and the Oneida Lowlands and Mohawk River valley (Fig 1). There are few antecedents in the area and it is likely that JCI populations moved into the region from elsewhere in Northern Iroquoia during the late fourteenth to early fifteenth centuries [11]. Many JCI sites were situated on defensive landforms and surrounded by earthworks. JCI populations may have experienced coalescence in a manner similar to populations to the north and south though the trend towards larger villages was not uniform across the region [12]. Social network analyses based on the designs incised and/or stamped on pottery vessel collars suggest that JCI occupied liaison brokerage [13] positions in regional networks [6]. As liaison brokers, JCI mediated interactions between northern (Ontario) and southern (Oneida Lowlands and Mohawk River) network components.

Two overarching models have been identified concerning the effects of brokerage positions in social networks that have been glossed as individualist and collectivist perspectives [14]. According to individualist models, actors occupying brokerage positions may accrue social capital positively in societies that value personal advancement and economic success [13,15–17]. However, in more collectively-oriented, and especially non-Western societies such as those of Northern Iroquoia, brokers may be viewed with suspicion or mistrust [14,17,18]. Archaeological applications of social network analysis have identified differences in the ways that the intermediate position of brokers has affected the historical development of regional settlement dynamics [6,14,19].

In addition to formal network analyses, distributions of artifacts also suggest important roles for JCI in regional interactions. Wonderley [20] analyzed distributions of certain effigy pipes that are most prevalent on JCI sites but also occur in smaller numbers on sites in the Oneida lowlands and Mohawk River valley. He suggested that this pattern reflects an interaction sphere consisting of “peaceful interaction of pipe-smoking representatives of the various communities,” in diplomatic activities ([20], p. 232). More recently Jones et al.’s [21] geochemical analysis of steatite beads (also see [22,23]) suggested that beads found on north shore Iroquoian sites originated in Jefferson County leading them to suggest that JCI were “engaging in processes of interaction and alliance-building with ancestral Huron-Wendat populations during the late 15th century” ([21], p. 513).

While it is clear that JCI were important components of regional networks, serving as liaison brokers between communities in Ontario and New York, there remains much to be learned about how and why they came to play this role. What factors led to the JCI serving as liaison brokers in fifteenth-century AD northern Iroquoia? We suggest that the location of these sites at *both* a physiographic boundary and a cultural divide between nascent Haudenosaunee and Wendat confederacies is one possible factor. Archaeological investigations of frontiers have generally focused on colonial and nation-state building contexts [24]. Only recently have analyses examined frontier settings in collectively-oriented societies (e.g., [6,14]). Jefferson County’s location is one of two overland routes between southern Ontario and New York, the other being on the west shore of Lake Ontario crossing the Niagara River. This strategic location—the lands that connect Lake Ontario to the St. Lawrence River Valley-Atlantic corridor—was a critical trade artery in the later era of European contact [25]. It thus stands to reason that this was also a strategic geographical location for Iroquoian peoples prior to European contact.

Previous pan-Iroquoian network analyses have been based on a large matrix of Brainerd-Robinson (BR) similarity coefficients calculated from site-specific counts of decorative motifs stamped and/or incised on the collars of pots [5, 6, 26–28]. Collars are thickened bands of clay that circle a pot’s rim extending up to several centimeters down from the lip, that served as highly visible decoration platforms. The decorations are interpreted as signals of traits of the women who made/used the pots [27]. These signals probably reflected women’s political activities among other traits [5]. Previous analyses have demonstrated that networks based on this matrix track regional settlement and socio-political trends and structures [5,27].

BR is a city block similarity coefficient that was developed specifically to assess the similarity of archaeological artifact assemblages [29–32] and has been widely used for that purpose in northeastern North America (e.g., [33]) and elsewhere (e.g., [34]). As a city block metric, the differences in percentages of categories between assemblages are totaled ([32], p. 233). This total is subtracted from the maximum possible value of 200 to create the similarity coefficient. While originally developed as a tool for chronological ordering of archaeological sites [29,30], the coefficient has been used as a measure of social interactions since the mid-twentieth century (e.g., [35,36]), and it is widely used in archaeological social network analyses as a measure of social interaction [37].

**Fig 2:**
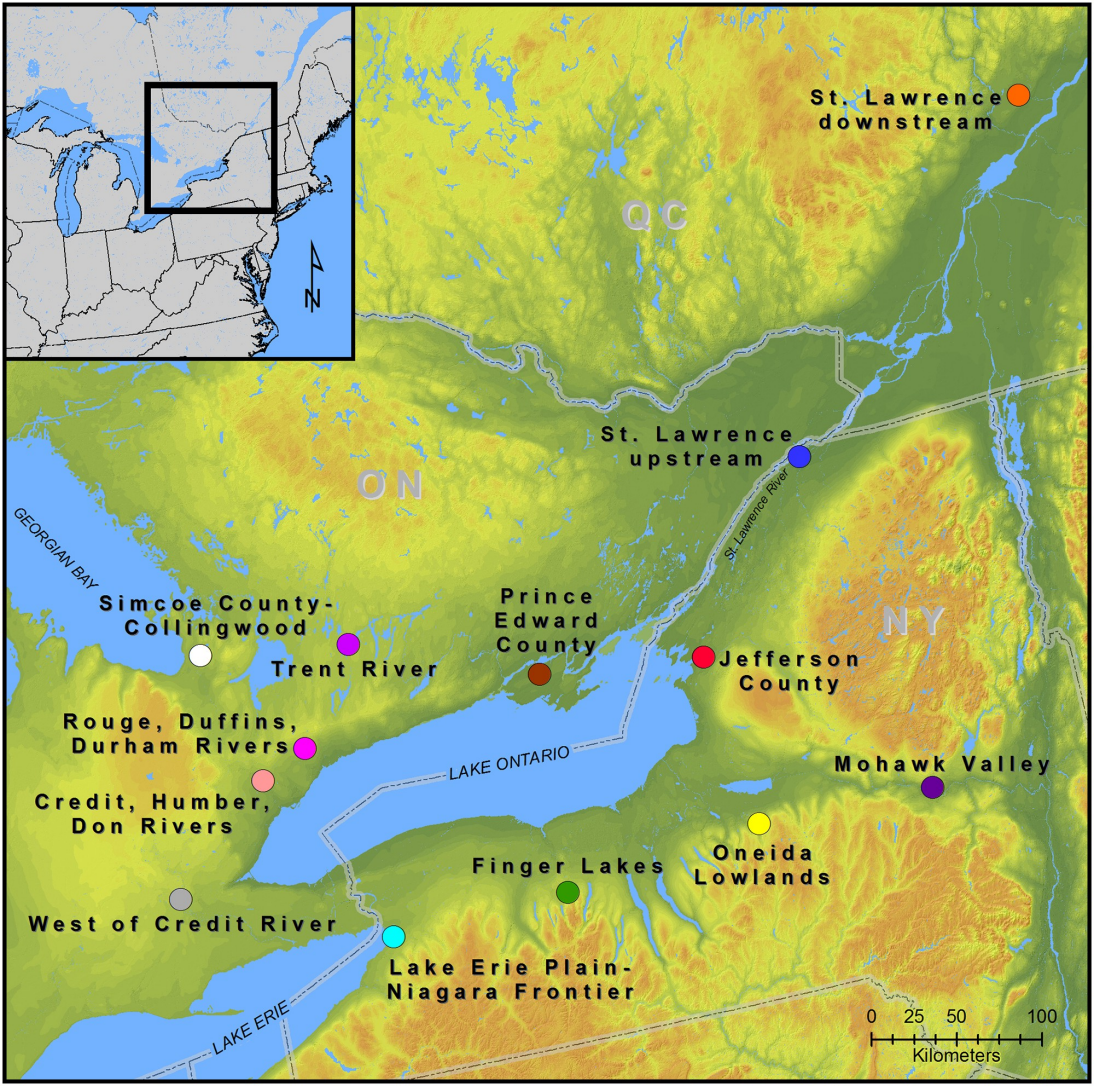
Map showing site cluster geographic centroids.

Northern Iroquoian settlement patterns display discrete clusters of village sites. These clusters were linked with overland trails that in some cases included segments of water-based travel. The overland trails were well established (e.g., [38,39]). Recent analysis of Haudenosaunee site location preferences, for example, identified proximities to overland trails as a consistently important factor in all models [40]. As suggested by Williamson and Snow [41] the specific terminal points of trail branches changed as village locations and other factors varied through time, but that the main branches of the trails probably had great antiquity. They suggest the trail networks were the symbolic backbone of northern Iroquoia. However, the historical record of overland trails is incomplete, and it cannot be used to determine overland trail distances between village clusters. As a result, for our analyses we performed a least cost path (LCP) analysis using Tobler’s Hiking Function. Because paths between all specific site pairs in two geographical clusters would be largely redundant and development of a matrix of LCPs between all individual sites would be time prohibitive, we developed a matrix based on single points representing the geographical center of each of the 13 clusters (hereafter centroids; Fig 2). While distances between specific sites of two clusters would be longer or shorter than distances between cluster centroids, given the size of northern Iroquoia, these differences in distance are negligible in terms of the hypothesis we test in the present analysis. To create an equivalent matrix of BR values, the means of site-level BR values were calculated between each cluster. Details for the LCP and BR calculations are described in the Materials and Methods section.

The previous analysis identifying JCI as occupying a brokerage liaison position in fifteenth-century AD northern Iroquoia was based on several network measures, including network fragmentation, node flow betweenness centrality, and edge betweenness centrality using both binarized and weighted graphs as appropriate to the particular measure [6]. To assess the brokerage position of geographic clusters for the current analysis, we used the potential brokerage measure for weighted graphs introduced by Peeples and Haas [14] based on Gould and Fernandez’s [13] measure for binary graphs and calculated using the R-script Peeples and Haas developed for their analysis of the North American Southwest. Prior to brokerage calculations, the LCP matrix was transformed into a similarity matrix by dividing each cell by the largest LCP value and subtracting the result from one. Potential brokerage was calculated for the mean BR site cluster and LCP centroid similarity matrices for each of three time spans, AD 1400–1500, AD 1450–1550, and AD 1500–1600, with 50-year overlaps as in our earlier analyses [5,6,28] to take into account any uncertainty in the chronological placement of individuals sites.

We use three metrics to test the hypothesis that the geographical location of the JCI was an important factor leading to their brokerage role in regional interaction networks. First, if geographic location was important to the development of the JCI brokerage we would expect a majority of overland trails to cross the headwaters of the St. Lawrence River where the JCI were located. Second, if distance was important in determining interrelationships between village clusters, we would expect significant, negative correlations between mean BR values and LCP and geodesic distances between centroids. Third, if geographic location was important in determining brokerage development, we would expect there to be positive correlations between potential brokerage values [14] calculated from BR and LCP similarity matrices. The results indicate that the hypothesis cannot be rejected.

## Results

**Fig 3:**
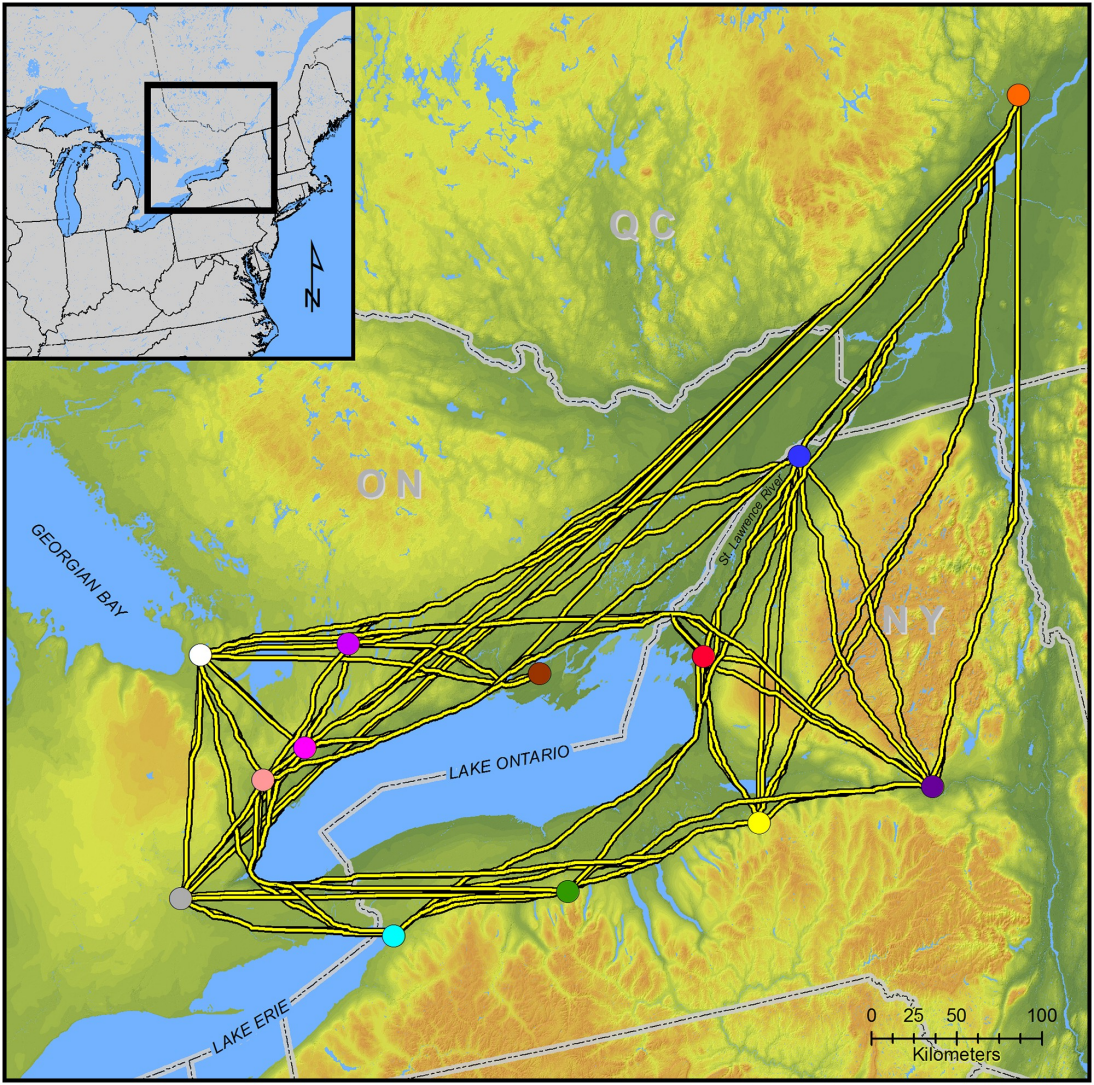
Least cost paths between geographic cluster centroids.

**Fig 4:**
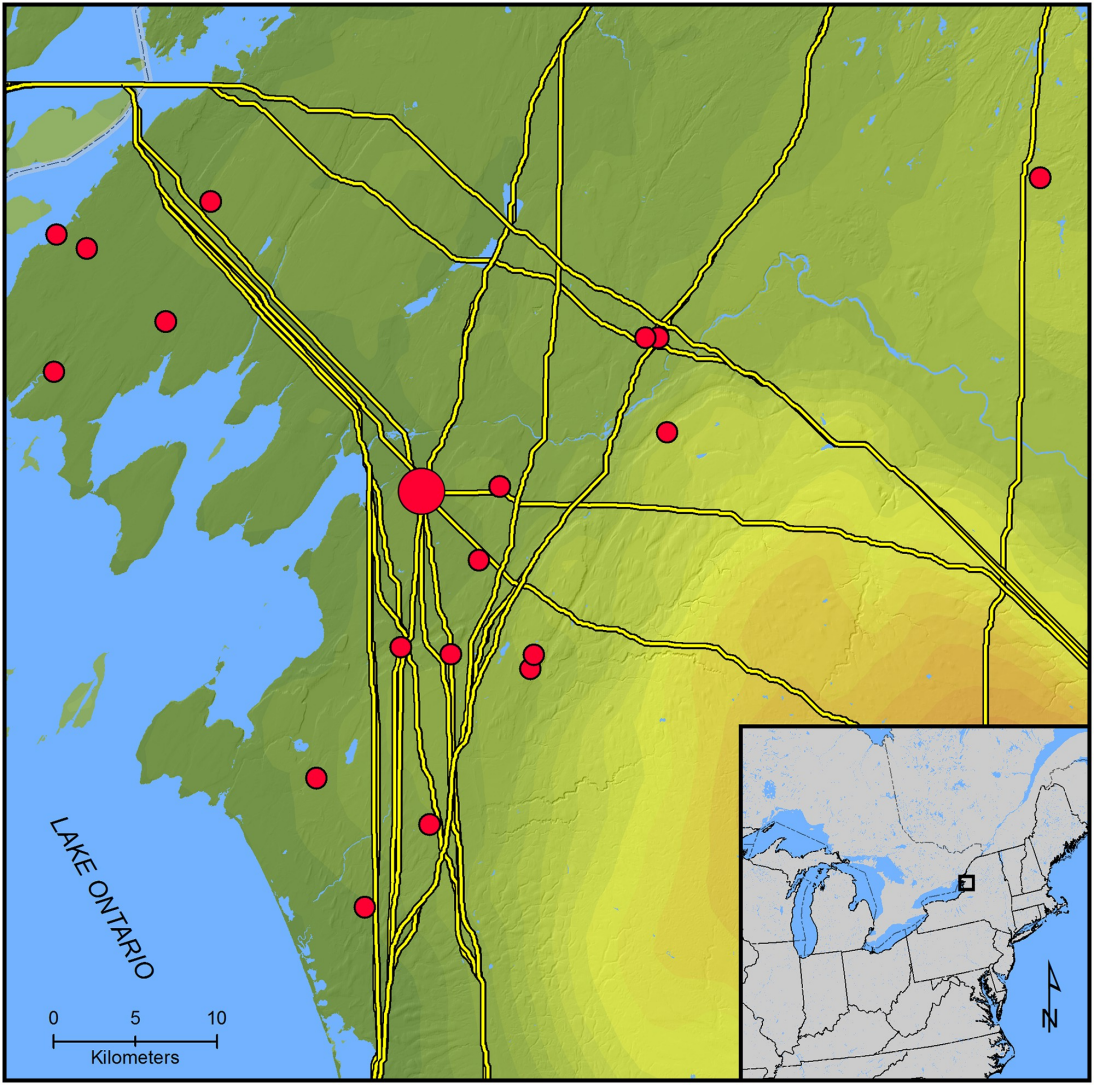
Jefferson County site locations and least-cost paths. The large dot is the geographic centroid, while the small dots are site locations.

The LCP results are illustrated in Fig 3. As an initial assessment of the potential for geographic position to affect JCI network brokerage positions we calculated the percentage of LCPs that crossed the headwaters of the St. Lawrence River as opposed to the Niagara River (Table 1). The majority of LCPs from the Mohawk River and Oneida Lowlands centroids to Ontario centroids cross the headwaters of the St. Lawrence River. Similarly, the majority of LCPs from eastern Ontario centroids to New York sites cross the St. Lawrence River headwaters. These results suggest the strategic location of JCI in regional interactions. Locations of JCI sites in our database relative to LCP segments are shown in Fig 4.

**Table 1:** Percentage of LCPs crossing the St. Lawrence or Niagara River from or to New York or southern Ontario.

Origin Centroid	St. Lawrence	Niagara
To 6 centroids in Ontario		
Finger Lakes	17	83
Jefferson County	100	0
Lake Erie Plain-Niagara Frontier	0	100
Mohawk River	83	17
Oneida Lowlands	67	33
To 5 centroids in New York		
Credit, Humber, Don Rivers	20	80
Prince Edward County	80	20
Rouges, Duffins, Durham Rivers	60	40
Simcoe County-Collingwood	60	40
Trent River	60	40
West of Credit River	20	80

Previous analyses [5,6,28,42] have shown that geodesic distances have limited effect on site-level BR coefficients, generally accounting for less than 30% of BR variation. However, QAP correlation of centroid BR similarity and geodesic or LCP distance matrices resulted in significant, moderate to high negative correlations (Table 2). QAP regressions of geodesic or LCP distance on BR similarity matrices resulted in coefficients of determination >0.300. These results indicate that LCP distances have moderate effects on average BR values between geographical clusters. The correlations and coefficients of determination increase in the AD 1500–1600 time span indicating increased social distance with increased spatial distance following the dispersal of JCI.

**Table 2:** QAP correlation and regression results of geodesic and LCP centroid distances on average BR similarity values between geographic clusters.

Graphs	Correlation	p-value	r^2^	p-value
AD 1400–1500 geodesic	‒0.626	0.001	0.392	0.002
AD 1450–1550 geodesic	‒0.620	0.001	0.384	0.001
AD 1500–1600 geodesic	‒0.711	0.001	0.506	0.001
AD 1400–1500 LCP	‒0.685	0.000	0.470	0.001
AD 1450–1550 LCP	‒0.634	0.000	0.402	0.001
AD 1500–1600 LCP	‒0.732	0.000	0.537	0.001

**Fig 5:**
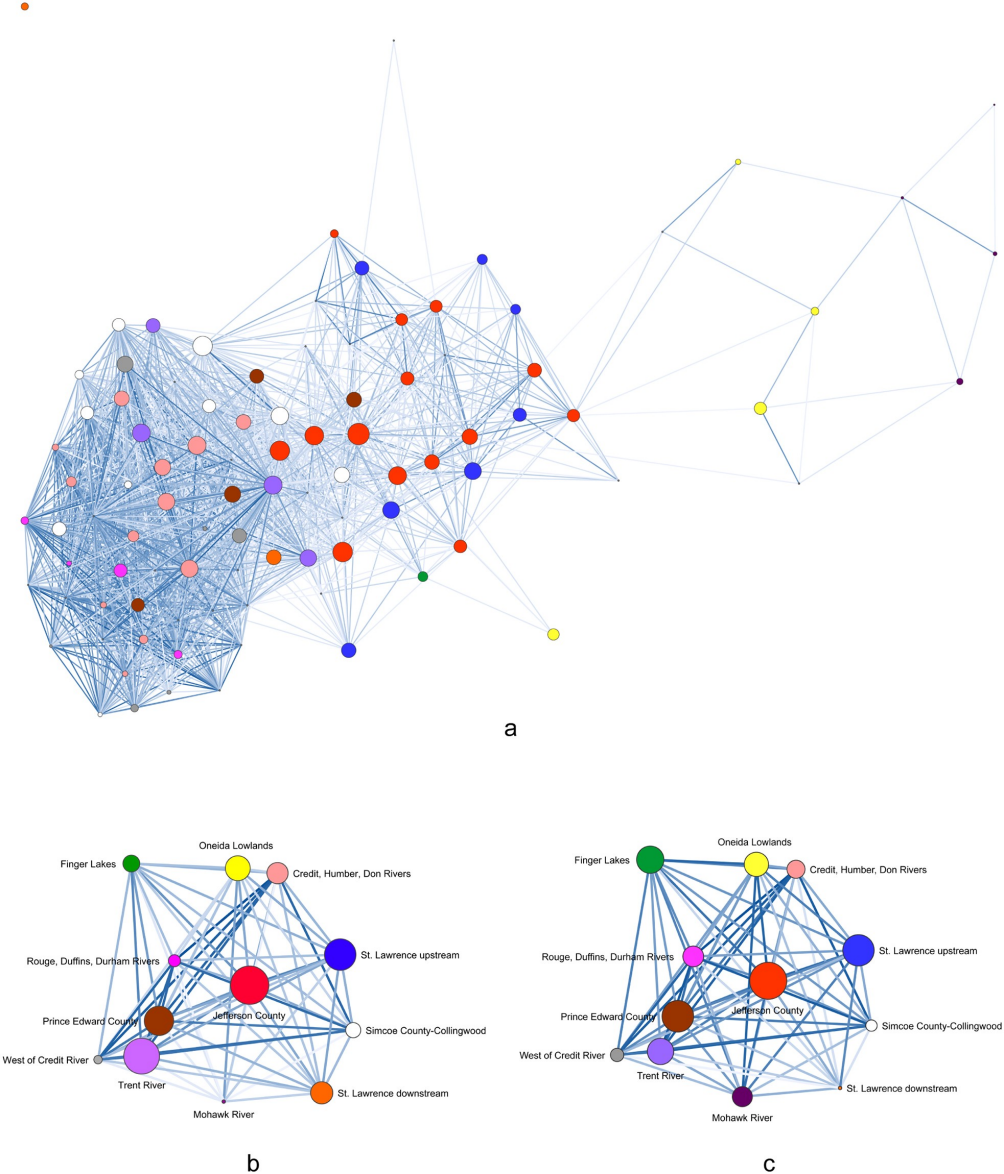
AD 1400–1500 network visualizations. (a) BR site-level network with threshold of ≥ 0.50 and spring embedder layout, (b) weighted BR centroid network with metric multidimensional scaling (MMDS) layout, (c) weighted LCP similarity centroid network with MMDS layout. Node size based on potential brokerage scores.

**Fig 6:**
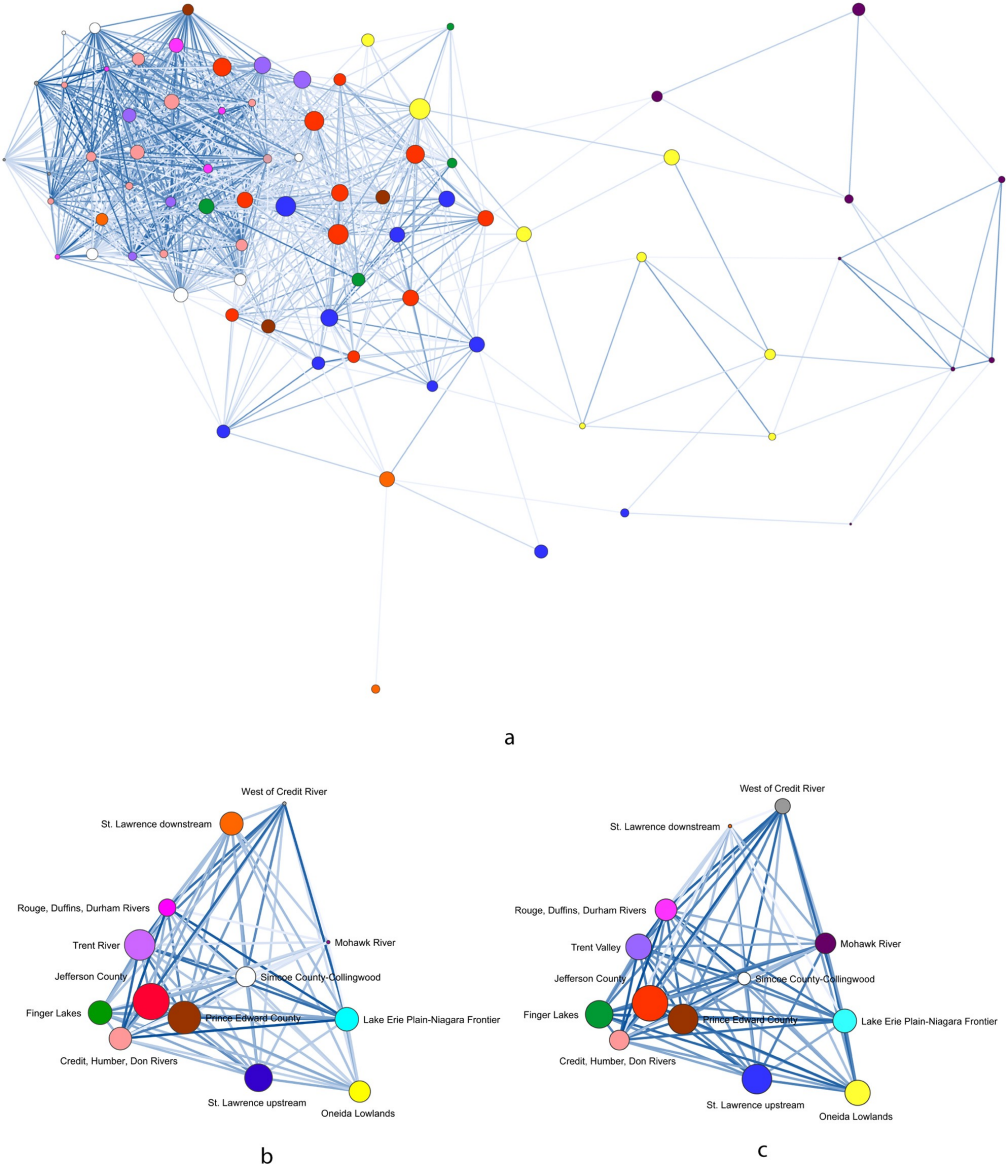
AD 1450–1550 network visualizations. (a) BR site-level network with threshold of ≥0.50 and spring embedder layout, (b) weighted BR centroid network with MMDS layout, (c) weighted LCP similarity centroid network with MMDS layout. Node size based on potential brokerage scores.

**Fig 7:**
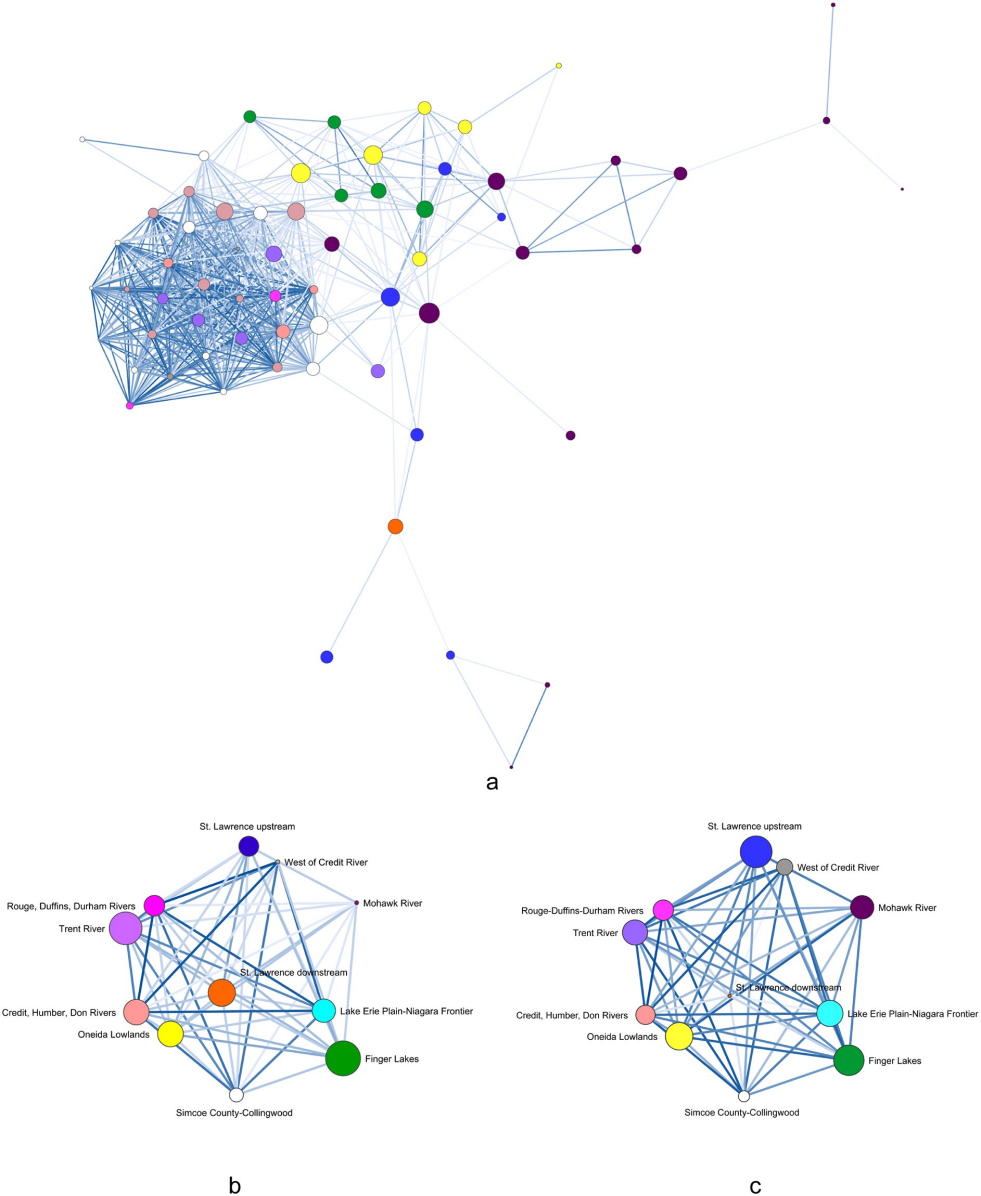
AD 1500–1600 network visualizations. (a) BR site-level network with threshold of ≥0.50 and spring embedder layout, (b) weighted BR centroid network with MMDS layout, (c) weighted LCP similarity centroid network with MMDS layout. Node size based on potential brokerage scores.

We calculated Peeples and Haas’ [14] brokerage measure on the BR matrices of individual sites for each 100-year time span. JCI sites account for 60-percent and 50-percent of the 90^th^-percentile of potential brokerage scores, in the AD 1400–1500 and AD 1450–1550 graphs, respectively (Table 3). No single geographic cluster accounts for more than 29 percent of the 90^th^ percentile in the AD 1500–1600 graph. These results are consistent with the previous analysis based on multiple network measures that identified JCI as occupying liaison brokerage positions in pan-Iroquoian social signaling networks [6]. These results are illustrated in Figs 5a–7a with node size based on potential brokerage values.

**Table 3:** Percentage of individual sites in the 90^th^-percentile of potential brokerage scores [14] by geographic cluster for weighted 100-year graphs.

Geographic Group	AD 1400–1500	AD 1450–1550	AD 1500–1600
Credit, Humber and Don Rivers	10.0	0.0	0.0
Finger Lakes	0.0	0.0	0.0
Jefferson County	60.0	50.0	—
Lake Erie Plain-Niagara Frontier	—	0.0	29.0
Mohawk River	0.0	0.0	14.0
Oneida Lowlands	0.0	12.5	29.0
Prince Edward County	0.0	0.0	—
Rouge, Duffins, Durham Rivers	0.0	0.0	0.0
Simcoe County-Collingwood	10.0	0.0	14.0
St. Lawrence downstream	0.0	0.0	0.0
St. Lawrence upstream	0.0	25.0	14.0
Trent River	20.0	12.5	0.0
West of Credit River	0.0	0.0	0.0

We next calculated Peeples and Haas’ [14] potential brokerage measure on the BR and LCP similarity centroid matrices. Several archaeological studies of regional interactions have applied network measures to LCP matrices (e.g., [43,44]) or other likely travel routes (e.g., [45]). Network measures are also used in in ecological studies of animal movements (e.g., [46]). These analyses often use measures of node centrality and other standard network measures to assess potential or actual interactions. Here we are interested in how geographical location may have determined in part the brokerage positions of site clusters. We reasoned that if geographical location was a significant factor in determining where there is a potential for brokerage to arise, then the total potential brokerage scores calculated from BR and the LCP similarity centroid matrices should be positively correlated.

Total BR and LCP similarity standardized brokerage scores for each centroid are presented in Table 4 by 100-year graph. Two results are presented for the AD 1400–1500 and AD 1500–1600 time spans. For AD 1400–1500, brokerage was calculated without the Lake Erie Plain-Niagara Frontier, for which there are no sites in our dataset. In the second the scores were calculated with zeros entered for all Lake Erie Plain-Niagara Frontier cells in the BR matrix resulting in the BR2 vector. We reasoned that even if no sites were present, the Lake Erie Plain-Niagara Frontier should still be taken into account in the LCP similarity calculations. The same process was followed for the AD 1500–1600 time span, with Jefferson County and Prince Edward County treated as missing values in the first set and with zeros entered into the BR matrix cells for the second (BR2). In both cases the full LCP total potential brokerage score vector (LCP2) was used in the correlation calculations with the BR2 vectors.

**Table 4:** Standardized brokerage scores for weighted 100-year average BR and LCP graphs by geographical cluster.

Geographic Cluster	AD 1400–1500		AD 1400–1500		AD 1450–1550		AD 1500–1600		AD 1500–1600	
	BR	LCP	BR2	LCP2	BR	LCP	BR	LCP	BR2	LCP
Credit, Humber and Don Rivers	0.077	0.109	0.071	0.148	0.103	0.148	0.083	0.121	0.070	0.148
Finger Lakes	0.048	0.248	0.045	0.288	0.115	0.288	0.**156**	0.298	**0.132**	0.288
Jefferson County	**0.250**	**0.457**	**0.231**	**0.482**	**0.268**	**0.482**	―	―	0.000	**0.482**
Lake Erie Plain-Niagara Frontier	―	―	0.000	0.211	0.113	0.211	0.07	0.222	0.059	0.211
Mohawk River	0.000	0.134	0.000	0.159	0.000	0.159	0.002	0.178	0.002	0.159
Oneida Lowlands	0.105	0.196	0.097	0.241	0.093	0.241	0.085	0.244	0.072	0.241
Prince Edward County	0.146	0.333	0.135	0.339	0.217	0.339	―	―	0.000	0.339
Rouge, Duffins, Durham Rivers	0.025	0.144	0.023	0.179	0.061	0.179	0.055	0.135	0.046	0.179
Simcoe County-Collingwood	0.039	0.043	0.036	0.059	0.081	0.059	0.025	0.040	0.021	0.059
St. Lawrence downstream	0.084	0.000	0.077	0.000	0.109	0.000	0.095	0.000	0.080	0.000
St. Lawrence upstream	0.169	0.328	0.156	0.332	0.156	0.332	0.051	**0.325**	0.043	0.332
Trent River	0.217	0.231	0.200	0.251	0.190	0.251	0.137	0.203	0.116	0.251
West of Credit River	0.012	0.059	0.011	0.088	0.000	0.088	0.002	0.085	0.002	0.088
Correlation	0.757		0.687		0.749		0.353		-0.084	
Regression R^2^	0.573		0.471		0.561		0.124		0.007	
Permutation p-value	0.005		0.007		0.003		0.294		0.786	

The absence of sites in our dataset from the Lake Erie Plain-Niagara Frontier for the AD 1400–1500 time span and the single site for the AD 1450–1550 time span reflect the archaeological record [47,48]. Settlement patterns appear to have been dispersed in this region during the fifteenth-century AD, and only a few small village sites have been identified. This contrasts with contemporaneous settlement patterns in Jefferson County, where many village sites have been recorded and partially excavated [9,49], and later sixteenth and seventeenth-century settlement of the Lake Erie Plain-Niagara Frontier when many large villages were present [47,48].

As can be seen in Table 4, Jefferson County has the highest potential brokerage value in each vector for the AD 1400–1500 and AD 1450–1550 time spans. For the AD 1500–1600 time span, the Finger Lakes has the highest potential brokerage values for the BR matrices. St. Lawrence upstream has the highest value for LCP vector. Permutation correlation and regression were run on each pair of vectors, with LCP as the independent variable. Results for the AD 1400–1500 and AD 1450–1550 graphs indicate moderate to high, positive correlations and R^2^ values well above 0.300. In all cases the results are significant (permutation p-values <0.05). For the AD 1500–1600 graphs, the results are low or negligible and not significant (permutation p-values >0.05). These results indicate that the null hypothesis of geographic location having no effect on brokerage can be rejected for the AD 1400–1500 and AD 1450–1550 graphs, but not for the AD 1500–1600 graphs.

As a separate test to confirm that the size of the vectors did not result in Type 1 or 2 errors, we calculated potential brokerage on the site-specific geodesic distance similarity matrix and ran permutation regressions on the standardized scores on the site-specific BR potential brokerage standardized scores. Calculating a site-level LCP matrix was prohibitive, but we assumed that like the centroid matrices (r = 0.965, p = 0.000), LCP and geodesic distances would be highly, positively correlated. The results indicate moderate, positive, significant correlations for the AD 1400–1500 and AD 1450–1550 time spans and negligible and not significant for the AD 1500–1600 time span (Table 5). These results suggest that the centroid vector sizes did not result in Type 1 or 2 errors.

**Table 5:** Correlations of site-level geodesic similarity and BR brokerage scores.

Statistic	AD 1400–1500	AD 1450–1550	AD 1500–1600
N	96	77	64
Correlation	0.415	0.518	0.013
Regression R^2^	0.172	0.268	0.000
Permutation p-value	0.000	0.000	0.919

In total, what these results indicate is that geographic location was a significant factor in determining the brokerage position of AD 1400–1500 and 1450–1500 village clusters. Therefore, we can reject the null hypothesis that geographical location did not affect the development of network brokerage positions. Jefferson County had the highest brokerage scores in the AD 1400–1500 and 1450–1500 graphs consistent with the earlier analysis that identified JCI occupying a liaison brokerage position in regional signaling networks. The JCI were at a nexus in the landscape [24] through which overland trails passed to crossings of the St. Lawrence River―the watercourse that served as a point of entry into northeastern North America—and routes of communication between communities and their member components and individuals. During the fifteenth century AD, the social landscape of Iroquoia was undergoing profound transformations. Between the mid-AD 1300s and late AD 1400s, cultural practices emerged in Iroquoia that emphasized integration with communities across the wider region [2,27]. However, after AD 1500, this pattern shifted to one of settlement aggregation, the intensification of conflict, and the formation of allied nations in both Ontario and New York [1,2,4]. After A.D. 1500 JCI populations dispersed from the region, presumably amalgamating with other, more coherent political entities [6–10]. The removal of JCI populations from the regional network contributed to increases in social distance, which is reflected in signaling patterns and lower average BR values between village clusters in the 1500–1600 period.

JCI groups traced their origins to other locations in northern Iroquoia, as well as possessing strong connections to downstream St. Lawrence populations. This may have contributed to their ability to maintain ties to multiple other groups in the network. However, the defensible and palisaded nature of most JCI settlements suggests a concern for defense during a period of increasing conflict and inter-societal tension. Among the nascent nations of the Haudenosaunee and the entirety of what would become the Wendat confederacy, these same processes led to the formation of stronger intra-group, bonding ties in the post-1500 period [5]. While brokerage positions may be desirable in the modern world system, in societies lacking strong political centralization, brokerage positions may be fraught and tenuous rather than advantageous [6,14]. In other parts of pre-Columbian North America, researchers have identified populations that occupied physiographic and demographic frontiers. Settlement in and around such boundaries tend to correlate with the simplification of social and political organizational structures with positions in social networks perhaps precluding increases in organizational complexity [19]. It is possible that occupying such a brokerage position prevented JCI populations from developing both strong internal cohesion and a cohesive, group-level alliance with either the Wendat or Haudenosaunee confederacies. Instead, *both* their geographic location at a strategic nexus in the landscape, and the diversity of ties that this position engendered ultimately resulted in their dispersal.

It is notable that JCI populations were key nodes in regional networks before, but not after, direct and sustained European contact. While it has been assumed that Iroquoian populations were eager to engage in and control connections to European, as well as Indigenous, trading partners [50,51] no other group chose to reoccupy this key position vis-à-vis overland trails and LCPs in the contact era.

## Conclusions

Previous analysis identified JCI as having occupied liaison brokerage positions in fifteenth-century AD pan-Northern Iroquoian social signaling networks, linking Iroquoians living on the north-shore of Lake Ontario and in the Oneida Lowlands and Mohawk River Valley [7]. This position for the JCI was identified using a series of network measures calculated from a large dataset of BR similarity coefficients. In the present analysis we confirmed this position using a single measure of potential brokerage for weighted graphs introduced by Peeples and Haas [14].

While our results are limited by sample size and chronological uncertainty, the present analyses suggest that the geographic location of the JCI contributed to the liaison brokerage positions they occupied in pan-northern Iroquoian social-signaling networks. There were, of course, other, so far unidentified, factors that contributed to the JCI liaison brokerage position in these networks. However, these analyses demonstrate we can state with some certainty it was the position of the JCI on the physical landscape, at a physiographic boundary, a frontier [19,24], that facilitated their network positions.

This assertion, that geographic location was critical to the emergence of JCI groups as brokers, taken in context with previous work highlighting the importance of social signaling behavior in generating political structures [5,6], does away with the either/or fallacies about the primacy of causal factors contributing to population dispersal. Rather, the results highlight the importance of both geographic (deterministic) and social (possibilistic) factors in fostering dynamic socio-political landscapes. Our results complement recent analyses of network brokerage in the North American Southwest [14,19] that indicate both the temporary nature of brokerage in collectively oriented societies and the importance of geographical locations in the development of regional network brokerage positions. Future analyses of network brokerage for such societies will benefit from explicitly comparative frameworks drawing on data from multiple regions.

## Materials and methods

LCP analysis was used to model a network of overland paths using ArcGIS 10.6. A destination-point feature layer, representing the geographic center of each of the 13 site clusters (centroid) used in previous analyses [6], was created using the Mean Center tool (Fig 2). This layer was projected into the North America Lambert Conformal Conic coordinate system. One-hundred and thirty-nine tiled 30-meter resolution digital elevation models (DEMs) were downloaded from the United States Geology Survey (USGS) National Map website. These DEMs, comprising the study area extent, were combined to create a singular raster file by using the Mosaic to New Raster tool. Prior to mosaicking, the original DEMs were checked for negative values and re-projected into the same coordinate system applied to the destination layer. If negative values found, all values less than zero were changed to zero using the Raster Calculator tool.

Lake Ontario was designated as a barrier to travel, requiring further processing of this raster dataset. A rectangular graphic encompassing the study area raster was digitized and converted to a polygon feature. A high-resolution bathymetry and shoreline raster of Lake Ontario was downloaded from the National Oceanic and Atmospheric Administration (NOAA) website and converted to a polygon. A polygon mask was constructed by joining these two polygons using the Union tool, and then deleting the inner Lake Ontario polygon. The Extract by Mask tool was applied to the study area raster, with this donut polygon selected as the mask. The resulting new raster dataset held NoData in the area formerly occupied by Lake Ontario 0-meter elevation values.

Tobler’s Hiking Function determines a person’s walking speed, accounting for the angle of slope. This function has been empirically tested [52] and in ArcGIS, estimates time (in hours) to cross a raster cell based on slope (in degrees) as calculated on a DEM. This function was used to model LCPs depicting the fastest in-bound routes to each of the 13 geographic group centroids (from every other centroid). A customized Tobler’s Hiking Function vertical factor table developed by Tripcevich [53] from data published by Tobler [54] was employed in the Path Distance tool along with the modified study area raster to produce cost distance and backlink rasters for each destination. These rasters were used as inputs in the Cost Path tool to generate the final LCP adjacency matrix with cells representing distances (km). As shown in Table 6, the average percent slope of these LCPs originating from each centroid range from 0.982 to 1.699, which obviated a need to calculate least-slope paths as an alternative solution to Tobler’s Hiking Function as has been done in other recent LCP analyses (e.g., [55]).

**Table 6:** Average percent slope for Tobler’s Hiking Function LCPs from one centroid to all other centroids.

Geographic Cluster	Average % Slope
Credit, Humber, Don Rivers	1.298
Finger Lakes	1.446
Jefferson County	1.179
Lake Erie Plain-Niagara Frontier	0.982
Mohawk River	1.699
Oneida Lowlands	1.495
Prince Edward County	1.150
Rouge, Duffins, Durham Rivers	1.207
Simcoe County-Collingwood	1.306
St. Lawrence downstream	1.040
St. Lawrence upstream	1.106
Trent River	1.174
West of Credit River	1.209

In previous analyses, graphs were created for 100-year time spans with 50-year overlaps to account for uncertainties in chronological placement [5,6,27]. For the present analyses three 100-year graphs were subject to analysis: AD 1400–1500, AD 1450–1550, and AD 1500–1600. JCIs came to inhabit northern New York in the early-to-mid fifteenth century AD and dispersed during the late fifteenth to early sixteenth centuries AD. The BR coefficients vary by site within and between geographic clusters. To create matrices of one BR value between geographic clusters to correspond to the LCP matrix, the mean of all BR values between all sites in two clusters was calculated. The number of sites in each cluster represented in the three graphs is presented in Table 7.

**Table 7:** Number of sites represented in geographical clusters by 100-year graph.

Geographic Cluster	AD 1400–1500	AD 1450–1550	AD 1500–1600
Credit, Humber, Don Rivers	18	10	3
Finger Lakes	3	4	5
Jefferson County	18	11	0
Lake Erie Plain-Niagara Frontier	0	1	9
Mohawk River	4	8	13
Oneida Lowlands	6	8	6
Prince Edward County	5	3	0
Rouge, Duffins, Durham Rivers	5	5	2
Simcoe County-Collingwood	12	6	12
St. Lawrence downstream	3	3	1
St. Lawrence upstream	9	10	6
Trent River	5	5	5
West of Credit River	8	3	2
Total	96	77	64

To test the null hypothesis that there is no relationship between geographic location and network brokerage position, we used a single measure of potential brokerage calculated from the BR and LCP similarity matrices. Following Peeples and Haas [14] total potential brokerage scores were standardized by dividing by the total number of nodes in each matrix. Peebles and Haas’s [14] R-script requires that matrix values be scaled between 0 and 1. The greatest distance in the LCP centroid matrix was 697 km. To transform the LCP distance measures to a similarity matrix, we divided all cells by 700 and subtracted the resulting values from one. For the site-specific geodesic distance matrices, we divided each cell by the largest distance in each matrix and then subtracted from one. BR values fall on a scale from 0 to 200, with 200 representing perfect similarity. The matrices were rescaled between 0 and 1 by dividing each cell by 200.

All statistical tests were carried out in UCINET 6.659 [56] and PAST 3.2 [57]. Given (1) the small size of the resulting vectors (12 for the AD 1400–1500 graph, 13 for the AD 1450–1550 graph, and 11 for the AD 1500–1600 graph), (2) the cases are in effect the population rather than a random sample of a population, and (3) as network measures independence cannot be assumed, we used permutation Pearson correlation and linear regression [58,59] to test the null hypothesis that there is no relationship between potential brokerage values based on LCP and BR. Quadratic Assignment Protocol (QAP) correlations and regressions were performed in UCINET for matrices with the default 5,000 random permutations for correlations and 2,000 for regressions. Node-based permutation correlations and regressions were performed with vectors in UCINET with the default 10,000 random permutations. We follow Hinkle et al.’s [60] rule-of-thumb for interpreting correlation coefficient size. Network visualizations were realized using visone 2.17 [61]. All matrices used in the statistical analyses are provided in the S1 Appendix.

## Supporting information

S1 Appendix Data used in statistical analyses. Centroid LCP distancesAverage site cluster BR matrixCentroid geodesic distancesSite-level BR matrix 1400–1500Site-level BR matrix 1450–1550Site-level BR matrix 1500–1600Geodesic distances 1400–1500Geodesic distances 1450–1550Geodesic distances 1500–1600Site-level brokerage 1400–1500Site-level brokerage 1450–1550Site-level brokerage 1500–1600.
(XLSX)
